# Traumatic Blunt Force Renal Injury in a Diseased Horseshoe Kidney with Successful Embolization to Treat Active Bleeding: A Case Report and Literature Review

**DOI:** 10.1155/2020/8897208

**Published:** 2020-07-24

**Authors:** Chonlada Krutsri, Pongsasit Singhatas, Preeda Sumpritpradit, Chunlaches Chaijareenont, Wit Viseshsindh, Tharin Thampongsa, Pattawia Choikrua

**Affiliations:** ^1^Division of Trauma Surgery and Surgical Critical Care, Department of Surgery, Faculty of Medicine, Ramathibodi Hospital, Mahidol University, Bangkok, Thailand; ^2^Department of Surgery, Faculty of Medicine, Ramathibodi Hospital, Mahidol University, Bangkok, Thailand; ^3^Division of Urology, Department of Surgery, Faculty of Medicine, Ramathibodi Hospital, Mahidol University, Bangkok, Thailand; ^4^Surgical Research Unit, Faculty of Medicine, Ramathibodi Hospital, Mahidol University, Bangkok, Thailand

## Abstract

**Background:**

Blunt force injuries in patients with preexisting kidney disease account for 19% of all kidney injuries, suggesting that diseased kidneys are more vulnerable than normal kidneys. When a horseshoe kidney (a rare anomaly: prevalence of 0.2%) is injured, treatment is challenging, especially when nonoperative management is desired. In high-grade blunt force normal kidney injury, nonoperative management has high succession rate (94.8%) with kidney-related complication (13.6%). Surgical reconstruction and preservation of a damaged horseshoe kidney is difficult because of variations in its vascular anatomy. We report successful nonoperative management of a blunt horseshoe kidney injury with active bleeding and review previous outcomes and complications. *Case Presentation*. A 57-year-old man had a head-on collision motorcycle road traffic accident. On arrival, blood pressure was 90/60 mmHg, pulse rate 140 bpm, and clear yellow urine output 200 ml. The patient was transiently responsive to fluid and blood component. Whole body computed tomography showed a high-volume retroperitoneal hematoma and multiple-lacerated lower pole of the kidney, compatible with preexisting horseshoe kidney disease with active contrast-enhanced extravasation from the accessory right renal artery. Embolization was performed. Renal function, transiently impaired after embolization, normalized on day 3. An infected hematoma found on day 7 was successfully controlled with antibiotics. His recovery was uneventful. At the 6-month follow-up, his serum creatinine level had returned to normal. The average age of blunt force horseshoe kidney injury is 31.75 years and occurred more common in male (87.5%).

**Conclusion:**

Diseased horseshoe kidneys are prone to injury even with low-velocity impact such as a road traffic accident speed < 15 km/h. Embolization is considered the first choice for management, with its high clinical success rate leading to less need for surgical repair. Not removing a hematoma is likely to result in complications. If embolization fails to stop bleeding, life-saving surgical exploration should be mandated.

## 1. Introduction

Blunt force kidney damage accounts for 1%–5% of all bodily injuries. Among kidney injuries, blunt force is responsible for 93.4%, making it ninefold more common than a penetrating injury [1, 2]. Preexisting kidney disease associated with a blunt force injury comprises 19% of all kidney injuries [3]. Blunt force injury to the more vulnerable diseased kidney should raise suspicion of the diagnosis when the injury is isolated and caused by low-velocity impact [3]. The most common presentation is gross hematuria, hemorrhagic shock class 2-3 based on Advanced Trauma Life Support (ATLS) principle with a retroperitoneal hematoma, and/or flank pain. Currently, a high-grade blunt force normal kidney injury can be managed nonoperatively (i.e., hemodynamic monitoring, embolization, and endovascular stent) with a high success rate of 94.8%, leading to a decreased nephrectomy rate of 5.4% [2]. A kidney-related complication (UTI, abscess, or hematuria) from nonoperative management rate is 13.6% which is not significantly different from immediate operation patients [4].

A horseshoe kidney is a rare anomaly with an overall prevalence of 0.2% with high prevalence in male to female (ratio 2 : 1) [5]. Blunt force injury to this anomalous kidney is challenging with regard to nonoperative management, especially if it is a high-grade injury with active bleeding. From the literature review of blunt force horseshoe kidney injury, the average age is 31.75 years and occurred more common in male (87.5%) [7–13].

According to the American Association for the Surgery of Trauma (AAST), the indication for surgery to treat blunt force injury of a normal kidney is the presence of unstable hemodynamics with a shattered, grade 5 kidney injury. In the presence of preexisting kidney disease, however, 57% of the indication for surgery depends on the hemodynamics and underlying parenchymal pathology, such as a tumor. For an injured diseased horseshoe kidney, surgical reconstruction and preservation of the damaged kidney is difficult because of variations in its vascular anatomy, which could lead to failed preservation of the kidney parenchyma.

We therefore report successful nonoperative management of a high-grade, actively bleeding horseshoe kidney that had been subjected to a blunt force injury. We also review the literature for outcomes and complications after various management protocols for treating horseshoe kidneys that have undergone a blunt force injury.

## 2. Case Report

A 57-year-old man had a head-on collision motorcycle road traffic accident to a street light pole in speed less than 40 km/h because of an acute stroke. His vitals on arrival are as follows: blood pressure 90/60 mmHg, pulse rate 140 bpm, and saturation 98%. The Advanced Trauma Life Support principle was performed, which revealed that he was transiently responsive and suffered grade 2 hemorrhagic shock. A focused assessment with sonography for trauma (FAST) was negative in all four quadrants. Physical examination found soft, no distension, no abdominal contusion, or peritonitis. A Foley catheter was inserted, which showed clear, yellow urine (200 ml).

**Figure 1:**
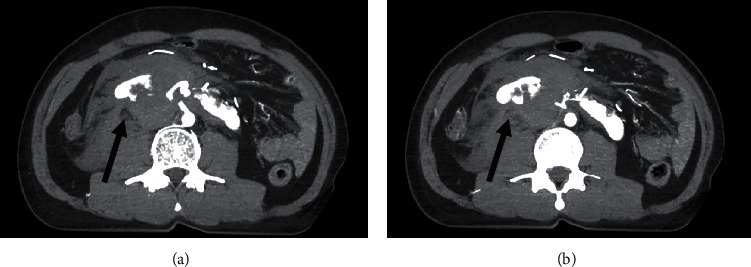
CT angiography in axial view, showing an active contrast extravasation from accessory right renal artery and retroperitoneal hematoma (a, b) (black arrow).

**Figure 2:**
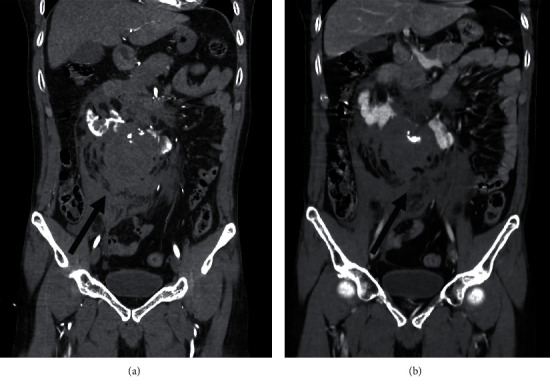
CT angiography in coronal view, showing an active contrast extravasation from accessory right renal artery and retroperitoneal hematoma (a, b) (black arrow).

The patient underwent whole body computed tomography (CT) scan by indication of blunt abdominal injury with hypovolemic shock grade 2, which revealed a retroperitoneal hematoma measuring 6.9 × 6.1 × 12.3 cm and multiple lacerations of the lower pole of the kidney. A CT brain (as part of whole body CT scan) shows acute ischemic stroke on MCA distribution. These findings were compatible with preexisting disease of his horseshoe kidney without a concomitant intra-abdominal organ injury or urinoma (Figures 1 and 2) There was active extravasation of contrast agent from the accessory right renal artery feeding into the lower pole of the right wing of the horseshoe kidney. This finding suggested that his transient responsiveness and grade 2 hemorrhagic shock were due to active retroperitoneal bleeding (Figures 1 and 2).

**Figure 3:**
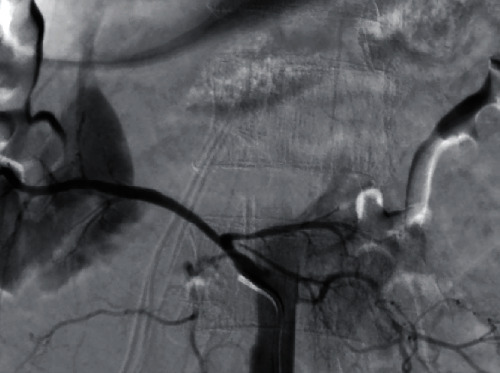
Angiogram before embolization. None of the eight patients was reported to suffer renal impairment. PR: pulse rate; BP: blood pressure; AAST: American Association for the Surgery of Trauma; PCD: percutaneous drainage.

Bleeding from the accessory right renal artery was managed successfully via endovascular coil embolization (Figure 3). The patient's renal function was impaired on postembolization day 1, with the serum creatinine at 2.33 mg/dL as compared to baseline serum creatinine at 1.32 mg/dL before undergoing whole body CT scan, which recovered to 1.58 mg/dL on postembolization day 3. The patient, however, developed a low-grade fever on postembolization day 7. He then underwent CT whole abdomen, which identified an infected hematoma from a finding of multiple air-fluid level with previous hematoma. It was successfully treated with an antibiotic, without surgery. The empirical antibiotic Ceftriaxone was used based on the clinical sepsis in an immunocompetent patient of intra-abdominal infection [6]. At the 6-month follow-up, his serum creatinine level had returned to normal (1.09 mg/dL). Informed consent to publish the case was obtained from the patient.

## 3. Discussion

We present a patient with active bleeding from the accessory right renal artery caused by blunt injury to a horseshoe kidney with preexisting disease. Schmidlin et al. reported that a blunt force injury to a kidney with preexisting disease is found in 19% of all kidney injuries, suggesting that diseased kidneys are more vulnerable to violence than normal kidneys [3]. The most common presentation of a patient with blunt force injury of the kidney includes gross hematuria, frank pain, and retroperitoneal bleeding or a hematoma. We should have elevated suspicion that a preexisting kidney injury exists when the injury is due to a low-impact velocity blow with no injury of the intra-abdominal organs [3].

CT is the gold standard method for grading kidney injury and revealing preexisting kidney disease. It provides more information than intravenous pyelography and gives information of concomitant injuries that might affect the choice of a treatment modality. Currently, even high-grade blunt force injury to a normal kidney injury can be managed nonoperatively (e.g., with embolization), achieving a high success rate of 94.8% and a low rate of 5.4% requiring nephrectomy [2]. Treating an injury of a horseshoe kidney with preexisting disease nonoperatively is challenging, especially when high-grade active bleeding is present because of variations in the kidney's anatomy, vascular blood supply, and parenchyma.

The indication for surgery of the kidneys with a preexisting disease is based on the patient's hemodynamics of shock classification 3 or 4 (base on ATLS) with no response to fluid and blood component and underlying kidney pathology, keeping in mind that it is not the same as the indication for injury of a normal kidney [3]. Hence, for this reason, nonoperative management should be mandated as the gold standard for addressing blunt force injury of a normal kidney as well as for a kidney with a preexisting disease if the patient's hemodynamics are stable and/or there is transient responsiveness of hemorrhagic shock classes 2 and 3.

A horseshoe kidney is a rare anomaly with an overall prevalence of 0.2%. It is important to preserve the injured kidney's parenchyma because there is no associated risk of cancer [7]. If surgery is necessary, one must be aware that surgical reconstruction and preservation of the parenchyma in a damaged horseshoe kidney is exceedingly difficult because of variation in the vascular anatomy. Sometimes, because of the variation of the artery feeding the kidney parenchyma, preservation of kidney parenchyma is likely to fail and nephrectomy cannot be avoided. Nonoperative management, such as embolization, is therefore preferable. A few types of nonoperative management of patients with horseshoe kidney disease who have experienced blunt force kidney damage have been proposed [7].

The present patient appears to be only the eighth reported case of blunt force injury to a horseshoe kidney and the fifth with nonoperative management. The details of our search of the English language literature on the various types of management and outcomes of blunt force-injured horseshoe kidneys are shown in Table 1 [7–13]. The average age of blunt force injury to a horseshoe kidney patients was 31.75 years, and this occurred more commonly in male patients (7/8, 87.5%). The average American Association for the Surgery of Trauma (AAST) kidney injury grade was 4. The most common presentation for this injury was gross hematuria and retroperitoneal bleeding, leading to hemorrhagic shock. Active extravasation of contrast medium, shown on CT angiography, occurred in five of the eight cases (62.5%). For the other three cases, it was not documented in two, and there was no contrast extravasation in one. Operative repair was required in three of the eight cases (37.5%).

There were three cases in which open operative repair was undertaken. In the first case, reported in 1998, the patient underwent operative repair because of hypotension and anemia [8]. In the second case, the patient was addressed surgically because of splenic rupture and an expanding retroperitoneal hematoma due to injury to a diseased horseshoe kidney [9]. During the surgery, the left-side peritoneal organs were medially rotated, revealing active bleeding from the left wing of the horseshoe kidney. The suprarenal aortic vessel was cross-clamped, and the left wing of the kidney was surgically removed [9]. The third case in which operative repair was indicated was addressed by bilateral transection of the horseshoe kidney. There had been active bleeding from the bilateral renal artery, leading to grade 3 hypovolemic shock. Embolization was not indicated because of the high risk of permanent kidney failure. Hence, medial visceral rotation was performed on both sides, after which the kidney parenchyma underwent suture repair [10].

The most common location of the injury among the eight reported patients was the lower pole (6/8 cases, 75%). This majority may be because the lower pole lies and fuses in the midline of the body, which is apt to be crushed to the lumbar spine during blunt force assault and is vulnerable to a shearing force. Embolization is the first choice of treatment of a blunt force injury to the normal kidney and, we believe, should be mandated for a diseased horseshoe kidney as well.

For management, there was a clinical success rate of 100% with embolization and endovascular stent placement. Complications occurred in three of the four (75%) patients with this treatment because the hematoma had not been removed. The complications included, respectively, an infected hematoma, retroperitoneal fluid collection, and retroperitoneal compartment syndrome even without increased bleeding [7, 11, 12]. Except for retroperitoneal compartment syndrome, these complications can be managed successfully with antibiotics and percutaneous drainage. Trottier et al. reported retroperitoneal compartment syndrome after endovascular stent placement for treating blunt force injury in a diseased horseshoe kidney [7]. Their patient had a systemic inflammatory response and renal failure secondary to the retroperitoneal compartment syndrome. The present hematoma had enlarged because of liquefaction and swelling of the retroperitoneum without increased bleeding. The clot was removed surgically, and the patient's condition dramatically improved. The retroperitoneal compartment syndrome may be caused by back bleeding from a lacerated arterial stump or parenchyma. Thus, when nonoperative management, such as embolization, is the first choice of treatment for a diseased horseshoe kidney with blunt force injury, we should be aware of that possible complication.

## 4. Conclusion

The number of reports of blunt force injuries in diseased horseshoe kidneys is limited because of the rare prevalence of the horseshoe kidney itself. Nevertheless, among those that are available, embolization had a high clinical success rate. Currently, nonoperative management, such as embolization, should be the first choice for management of blunt force injuries of a diseased horseshoe kidney. We should also be aware of the possibility of complications if the hematoma is not removed. If embolization fails to stop a patient's bleeding, surgical exploration should be mandated even though it is difficult because of the variation in the vascular anatomy of the horseshoe kidney.

## Figures and Tables

**Table 1:** Summary of reported treatment outcomes and complications of blunt force injuries of horseshoe kidneys.

Author (publication year)	Age (years)	Sex	PR (bpm)	BP (mmHg)	AAST (grade)	Signs and symptoms	Active extravasation of contrast medium	Management	Location of injury	Complication
Daudia (1998) [8]	25	Male	60	100/60	NA	Gross hematuria Anemia	NA	Open repair	Left lower pole	None
Legg (1998) [11]	49	Male	102	128/84	NA	Hemorrhagic shock grade 2 Anemia	Yes	Embolization	Left lower pole accessory renal artery	Retroperitoneal fluid collection
Trottier (2009) [7]	21	Male	Tachycardia (no number)	Hypotensive (no number)	4	Hemorrhagic shock grade 3	Yes	Endovascular with stent	Right polar artery arising from the common iliac artery	Compartment syndrome of the retroperitoneum
Dominguez (2010) [13]	16	Female	Stable (no number)	Stable (no number)	4	Kidney transection	No	Conservative	None	None
Molina Escudero (2011) [12]	25	Male	Stable (no number)	Stable (no number)	NA	Gross hematuria Hemorrhagic shock grade 3	Yes	Embolization	Lower branch of right renal artery	None
Paragi (2011) [9]	30	Male	Stable (no number)	Stable (no number)	NA	Expanding retroperitoneal hematoma Hypotension	NA	Open repair	Left wing of horseshoe kidney	Left subdiaphragmatic abscess
Cortese (2016) [10]	31	Male	140	80/50	4	Hemorrhagic shock grade 3	Yes	Open repair	Bilateral renal artery	None
Present case (2019)	57	Male	140	90/60	4	Hemorrhagic shock grade 2	Yes	Embolization	Lower branch of right renal artery	Infected hematoma
